# Estimation of sorghum seedling number from drone image based on support vector machine and YOLO algorithms

**DOI:** 10.3389/fpls.2024.1399872

**Published:** 2024-09-26

**Authors:** Hongxing Chen, Hui Chen, Xiaoyun Huang, Song Zhang, Shengxi Chen, Fulang Cen, Tengbing He, Quanzhi Zhao, Zhenran Gao

**Affiliations:** ^1^ College of Agriculture, Guizhou University, Guiyang, China; ^2^ Institute of New Rural Development, Guizhou University, Guiyang, China; ^3^ Institute of Rice Industry Technology Research, Guizhou University, Guiyang, China

**Keywords:** UAV, sorghum, seedling, SVM, YOLO

## Abstract

Accurately counting the number of sorghum seedlings from images captured by unmanned aerial vehicles (UAV) is useful for identifying sorghum varieties with high seedling emergence rates in breeding programs. The traditional method is manual counting, which is time-consuming and laborious. Recently, UAV have been widely used for crop growth monitoring because of their low cost, and their ability to collect high-resolution images and other data non-destructively. However, estimating the number of sorghum seedlings is challenging because of the complexity of field environments. The aim of this study was to test three models for counting sorghum seedlings rapidly and automatically from red-green-blue (RGB) images captured at different flight altitudes by a UAV. The three models were a machine learning approach (Support Vector Machines, SVM) and two deep learning approaches (YOLOv5 and YOLOv8). The robustness of the models was verified using RGB images collected at different heights. The *R^2^* values of the model outputs for images captured at heights of 15 m, 30 m, and 45 m were, respectively, (SVM: 0.67, 0.57, 0.51), (YOLOv5: 0.76, 0.57, 0.56), and (YOLOv8: 0.93, 0.90, 0.71). Therefore, the YOLOv8 model was most accurate in estimating the number of sorghum seedlings. The results indicate that UAV images combined with an appropriate model can be effective for large-scale counting of sorghum seedlings. This method will be a useful tool for sorghum phenotyping.

## 1 Introduction

Sorghum is among the five major cereal grains globally (Ritter et al., 2007; Motlhaodi et al., 2014), Sorghum growth (e.g., the number and size of seedlings and mature plants) is routinely monitored through manual measurements, which are less accurate and require substantial inputs in terms of labor, material resources, and time (Jiang et al., 2020). Modern agriculture urgently requires methods for the rapid assessment of crop emergence across large areas. Accurate estimates of crop populations, the number of plants in a stand, and evenness are essential for modern agriculture. Acquiring high-resolution remote sensing images using unmanned aerial vehicle (UAV) platforms allows for the rapid identification of seedling emergence and counting of plant numbers. The use of drones is a rapidly advancing technique in the field of agricultural observation (Maes and Steppe, 2019). Compared with low-altitude drone images, satellite remote sensing images are expensive to obtain and easily affected by weather factors (Dimitrios et al., 2018). Drones are inexpensive, can collect high-resolution images, and can carry a variety of sensors, such as red-green-blue (RGB), multispectral, and hyperspectral sensors. Therefore, the use of images collected by UAV can effectively solve the difficulty of obtaining crop phenotypic information (e.g., seedling identification and leaf area index (LAI) identification) from satellite images (Lin, 2008; Bagheri, 2017). Crop phenotype monitoring based on UAV images mainly uses image color segmentation (Zhao et al., 2021b; Serouart et al., 2022), machine learning (Lv et al., 2019; Ding et al., 2023), and deep learning (Qi et al., 2022; Zhang et al., 2023b) algorithms. The identification of crop seedlings through image color segmentation relies on color space transformation, employing threshold segmentation to differentiate between the foreground and background (Cheng et al., 2001) to extract the seedlings. Several studies have compared different data processing methods for images obtained by drones. For example, Gao et al. (2023) processed images of maize seedlings obtained by drones using color comparison, morphological processing, and threshold segmentation, and obtained accurate estimates of seedling numbers. Liu et al. (2020) extracted plant information from UAV images based on a threshold segmentation method combined with a corner detection algorithm, and obtained accurate estimates of the number of corn plants. Lei et al. (2017) used a threshold method to binarize images, followed by erosion and dilation processes, to optimize the recognition of cotton seedlings at 10 days post-planting in images captured by a drone at a flight altitude of 10 m. Image segmentation can be used to monitor crop growth indicators, but the height at which images are captured significantly affects the accuracy of image color segmentation (Liu, 2021). Threshold segmentation methods are susceptible to factors such as plant occlusion, weed growth, light intensity, and small target crops, and so their ability to separate the plants from the background is limited (Zhou et al., 2018).

In recent years, with the rapid development of big data technology and high-performance computers, machine learning technology has been widely used (Tan et al., 2022; Tseng et al., 2022). In traditional machine learning algorithms, features such as color and texture are extracted from RGB and multispectral images processed using different algorithms such as Nearest Neighbor, Random Forest, and Support Vector Machine (SVM) algorithms. Ahmed et al. (2012) used an SVM algorithm to distinguish six types of weeds and crops, and achieved an accuracy rate of 97%. Siddiqi et al. (2014) applied global histogram equalization to reduce the effect of illumination, and used an SVM algorithm for weed detection, achieving an accuracy of 98%. Saha et al. (2016) used an open-source dataset and applied an SVM algorithm to detect crop weeds, achieving an accuracy rate of 89%. Because machine learning features rely on manual extraction, in complex environments, particularly for small sample sizes and tasks with unclear local features, the learning efficiency is low, generalization is weak, and achieving satisfactory recognition results is challenging.

The availability of big data is promoting the rapid development of smart agriculture, and digital transformation has become an inevitable choice for agricultural modernization. Using deep learning technology to identify crops has become a research hotspot (Asiri, 2023; Feng et al., 2023). Compared with traditional machine learning algorithms, deep learning algorithms rely entirely on data, without human intervention, and use all of the information contained in the data for object detection. YOLO is a fast object detection algorithm using convolutional neural networks. It has the advantage of directly performing regression to detect objects in images (Redmon et al., 2016). Using the object detection model YOLOv5, Li et al. (2022) counted corn plants with an average accuracy (mAP@0.5) of 0.91. Su (2023) used YOLOv3, YOLOv4, and YOLOv5 models to detect rapeseed seedlings in images captured at different heights, and achieved the best detection performance using images collected at 6 m with a low degree of overlap. Korznikov et al. (2023) used YOLOv8 to identify plants of two evergreen coniferous species in remote sensing images, with an overall accuracy of 0.98. These examples show that the YOLO model has great potential in object detection. Among them, YOLOv5 is widely used due to its high detection accuracy and fast inference speed (Zhang et al., 2022). YOLOv8, as the most advanced version of the YOLO series, not only retains the advantages of the older version, i.e., good recognition accuracy and fast recognition speed, but also shows improved model performance. The sorghum seedlings in images collected by drones are present in a small area, have a high density, show severe overlapping, and are sometimes obstructed, and all of these factors contribute to false or missed detection. Therefore, it is of great significance to evaluate the suitability of the YOLOv5 and YOLOv8 models for the recognition and counting of sorghum seedlings in images collected by UAV.

In summary, machine learning and deep learning algorithms can process remote sensing image data with small targets and complex backgrounds, and make accurate predictions and decisions. Therefore, they are crucial methods for modern agriculture. In this study, we tested three different models for counting sorghum seedlings in UAV images captured at different heights. The objectives of this study were to: (1) combine a color vegetation index and maximum interclass variance (Otsu) method with an appropriate threshold for sorghum target recognition and image segmentation; (2) compare three models [a machine learning approach (SVM) and two deep learning approaches (YOLOv5 and YOLOv8)] to count the number of sorghum seedlings in the images; and (3) to compare and discuss the applicability of each method. The results of our study have potential applications for accurate field management.

## 2 Materials and methods

### 2.1 Study area and experimental design

**Figure 1:**
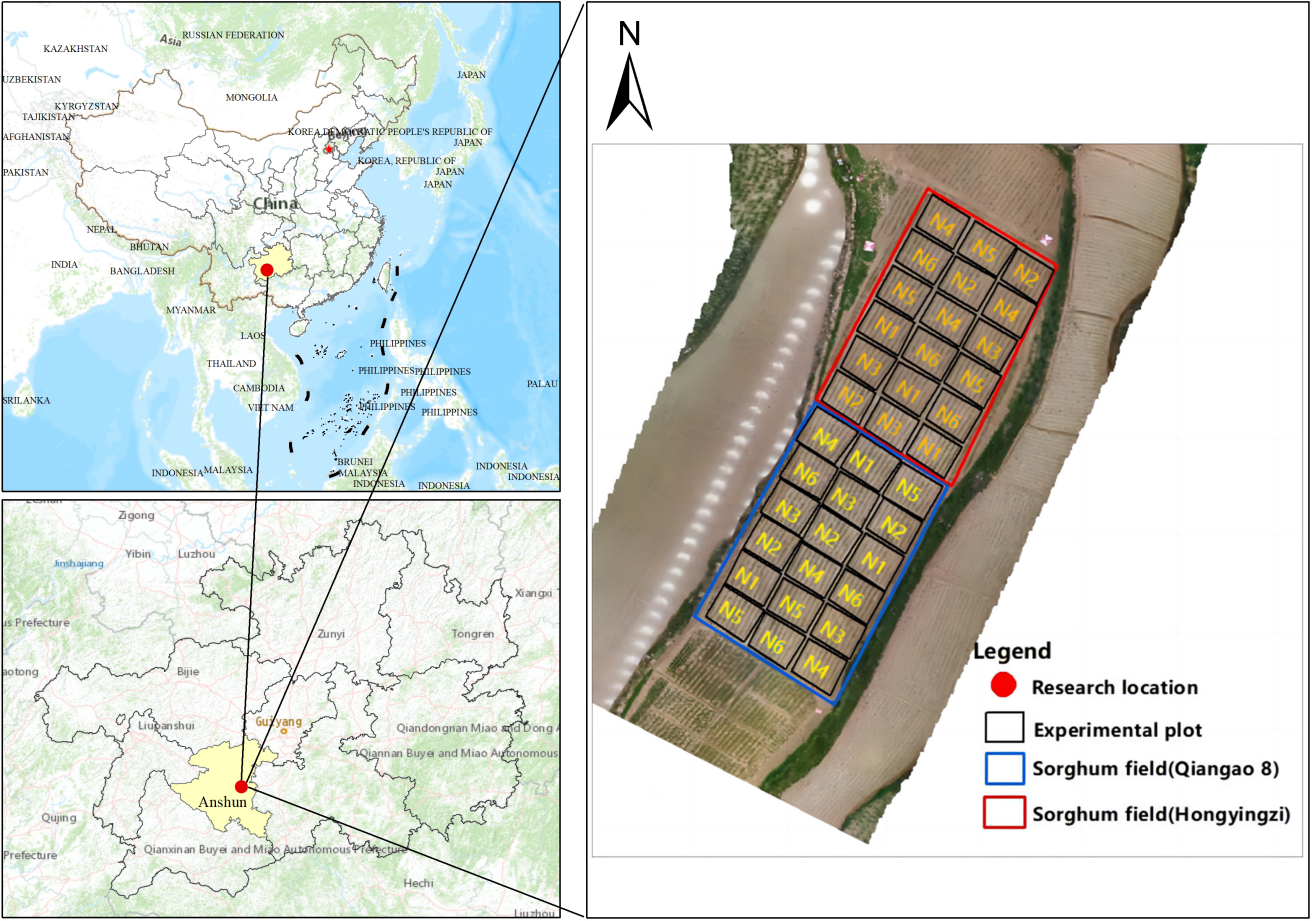
Location Map of the Study Area.

The research area was located in the village of Lianxing, near the town of Jichang, Xixiu District, Anshun City, Guizhou Province (105°44′–106°21′ E, 25°56′–26°27′ N). The sorghum varieties planted in the experimental area were Qiangao 8 and Hongyingzi. The experiment used a two-factor split-plot design, with variety as the main plot and fertilizer application as the secondary plot. The field experiment included six nitrogen concentration treatments, with nitrogen application ranging from 0 kg/hm^2^ to 300 kg/hm^2^ [including N1 (0 kg/hm^2^), N2 (60 kg/hm^2^), N3 (120 kg/hm^2^), N4 (180 kg/hm^2^), N5 (240 kg/hm^2^) and N6 (300 kg/hm^2^)]. Each experimental plot was 5 m long and 4 m wide, with a plot area of 20 m^2^ and a row spacing of 0.6 m. For each variety, there were three replicates of each nitrogen treatment. The seedlings were planted on April 16, 2023, and transplanting was carried out on May 7. The drone image data of the sorghum seedlings were collected on June 11. The experimental site is shown in **Figure 1**.

### 2.2 Data acquisition and preprocessing

To capture images, the lens in the drone was pointed vertically downwards and images were acquired at 2-second intervals. The flight altitudes were 15 m, 30 m, and 45 m, with a lateral overlap of 80% and a heading overlap of 70%. The camera parameters were as follows: DFOV 82.9°C, focal length 4.5 mm, aperture f/2.8, effective pixel of wide-angle camera sensor 12 million, image format TIFF, image type RGB. The collected images were spliced into orthophoto images using Photoscan software. The spliced orthophoto images were stored in TIFF format.

### 2.3 Statistical analysis

#### 2.3.1 Color feature analysis

In the visible light images of sorghum seedlings collected by the UAV, the soil was brown and the vegetation was green. To interpret UAV images, it is necessary to find a color feature that highlights the difference between the plants and the background, and then use threshold segmentation to successfully extract the target, in this case, sorghum plants. Commonly used plant extraction algorithms are segmentation methods based on vegetation color (Wang et al., 2019), In this study, we selected eight color indices that are widely used for analyses of UAV image data, namely the EXG index (Soontranon et al., 2014), the Cg index in the YCrCb color space (Lu et al., 2016), the EXR index (Zheng et al., 2017), the EXG-EXR index (Zheng et al., 2017), the GBDI index (Turhal, 2022), the NGBDI index (Song et al., 2022), the NGRDI index (Gitelson et al., 2002), and the S component in the HSV color space (Zhao et al., 2016). Based on analyses of color features and the ability of each index to extract the sorghum target, the optimal color indices were selected. The formula for each candidate color index is shown in **Table 1**.

**Table 1:** Color index list.

Color Vegetation Indices	Abbreviation	Formula
Excess green index (Soontranon et al., 2014)	EXG	2*g − r − b*
YCrCb–green difference index (Lu and Yang, 2016)	Cg	0.4*g −* 0.3*r −* 0.1*b*
Excess green minus excess red index (Zheng et al., 2017)	EXG-EXR	3*g −* 2.4*r − b*
Excess red index (Zheng et al., 2017)	EXR	1.4*r − g*
Green–blue difference index (Turhal, 2022)	GBDI	*g − b*
Normalized green minus blue difference index (Song et al., 2022)	NGBDI	(*g − b*)/(*g* + *b*)
Normalized green minus red difference index (Gitelson et al., 2002)	NGRDI	(*g − r*)/(*g* + *r*)
HSV–S-component (Zhao et al., 2016)	S	–

#### 2.3.2 Image segmentation based on Otsu threshold

Image segmentation algorithms extract and segment regions of interest based on the similarities or differences of pixel features, such as typical threshold segmentation, region segmentation, and edge segmentation. The Otsu method (Xiao et al., 2019) is a typical thresholding method that segments images based on the differences between pixels. According to the differences in image pixels, the image content is divided into target and background. Using statistical methods, a threshold is selected to maximize the difference between the target and background. Suppose the image size of the sorghum field is *M×N*, and the threshold value for separating the sorghum target from the background is *T*. Among them, the proportion of the number of pixels *N_0_* in the target area out of the total number of pixels in the image is denoted as *ω_0_*, and the average gray value is denoted as *μ_0_*. The proportion of the number of pixels *N_1_* of the background area out of the total number of pixels in the image is denoted as *ω_1_*, and the average gray level is denoted as *μ_1_*. The average gray level of the sorghum field image is denoted as *μ*, and the interclass variance is denoted as *S*. The larger the variance, the more obvious the difference between the target and background in the image, and the better the segmentation effect. The optimal threshold is the one that maximizes *S*. The specific implementation is described in the following sections.


(1) $$\omega_{0}=\frac{N_{0}}{M\times N}$$

(2) $$\omega_{1}=\frac{N_{1}}{M\times N}\;$$

(3) $$N_{0}+N_{1}=M\times N$$

(4) $$\omega_{0}+\omega_{1}=1$$

(5) $$\mu =\omega_{0}\times \mu_{0}+\omega_{1}\times \mu_{1}\;$$

(6) $$\text{S}={\text{ω}}_{0}{\left({\text{μ}}_{0}-\text{μ}\right)}^{2}+{\text{ω}}_{1}{\left({\text{μ}}_{1}-\text{μ}\right)}^{2}$$

#### 2.3.3 Machine learning - support vector machines

The SVM algorithm a generalized linear classifier that performs binary classification on data using supervised learning. Its decision boundary is a maximum-margin hyperplane that is solved for the learning samples. Compared with logistic regression and neural networks, SVM provides a clearer and more powerful approach for learning complex non-linear equations.

#### 2.3.4 Image marking

First, the puzzle software Photoscan was used to stitch the collected drone images into an orthophoto, and the stitched orthophoto was stored in TIFF format. The second step was to crop the RGB image obtained using ENVI5.3. This cropping operation eliminated the distortion and abnormal edge data generated during the stitching process, thereby reducing the impact on related algorithm design and model construction. The third step was to label the sorghum seedlings using labeling software Labelme. In total, 108 segmented images were opened for labeling, and 12,755 sorghum seedlings were labeled (each labeling box had two beads). The marked RGB image was stored in JSON format, and a script written using PyCharm was used to convert the JSON file into TXT format for storage of the dataset. The dataset was divided into a training set and a validation set (ratio, 2:1) is shown in **Table 2**.

**Table 2:** Partitioning the training set and validation set based on different criteria.

Height	Train Set	Val Set	Total
15 m	24	12	36
30 m	24	12	36
45 m	24	12	36
Total	72	36	108

#### 2.3.5 Deep learning algorithms (YOLOv5 and YOLOv8)

**Figure 2:**
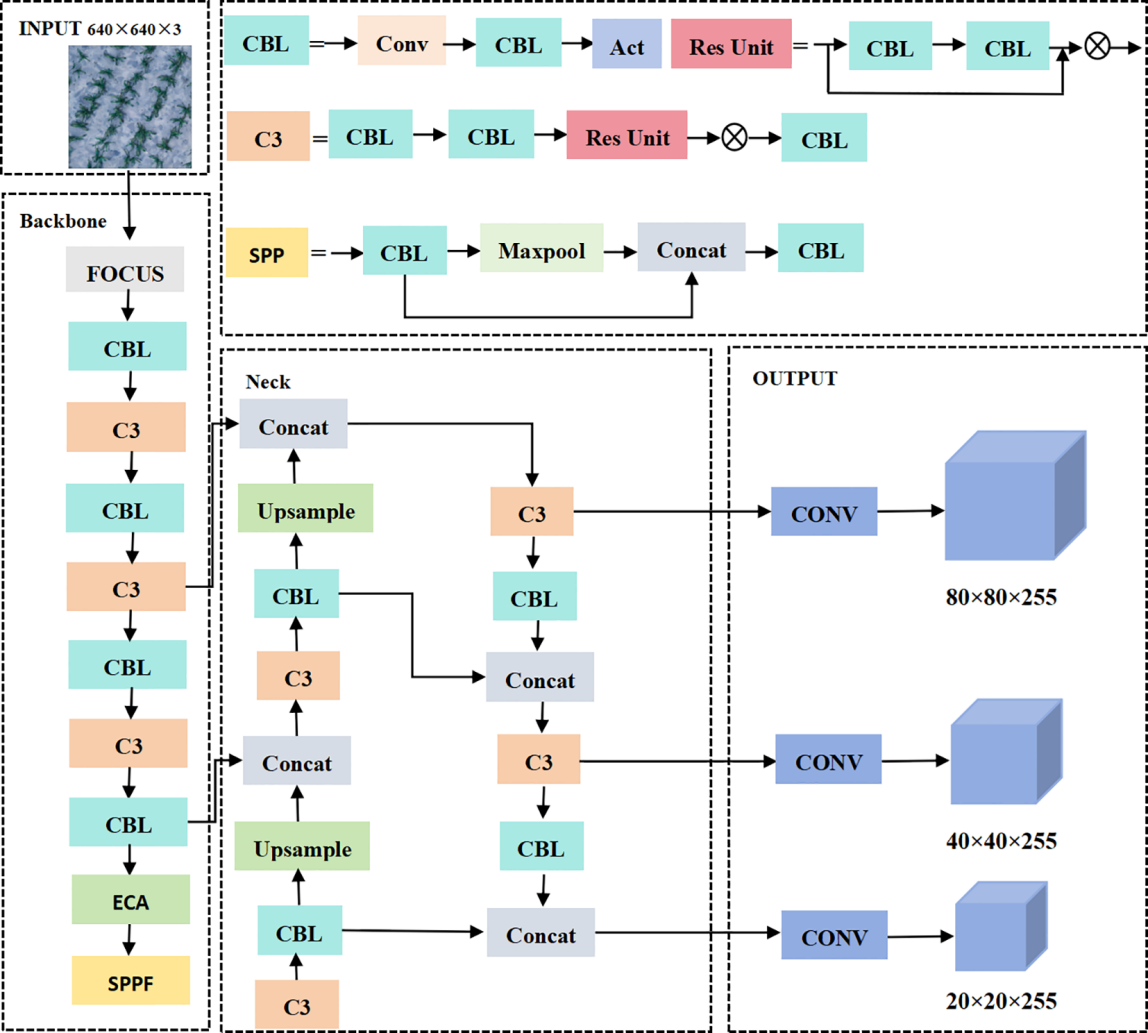
YOLOv5 model structure.

The YOLO series is a single-stage object detection algorithm proposed by Redmon et al. (2016). Essentially, the entire image is used as the input of the network and the target location coordinates, and category are the outputs. YOLOv5 is the most classic and stable version of the YOLO series. Its advantages in terms of maintaining detection accuracy are the smaller-weight files, shorter training time, and faster speed. The network structure model of YOLOv5 is shown in **Figure 2**.

**Figure 3:**
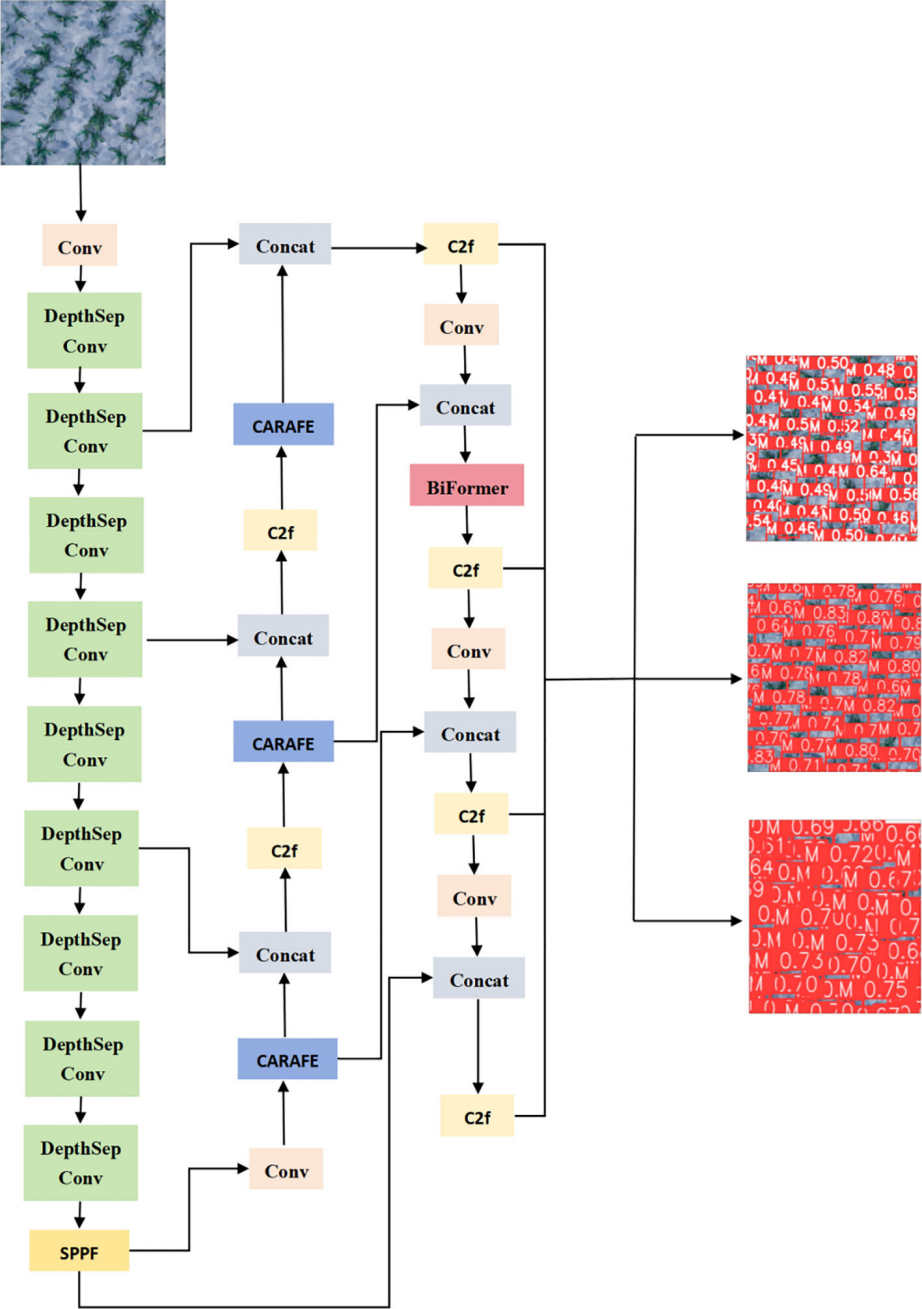
YOLOv8 model structure.

The YOLOv8 object detection algorithm inherits the thinking of YOLOv1 series, and is a new end-to-end object detection algorithm. The network structure of YOLOv8 is shown in **Figure 3**. In YOLOv8, the C3 backbone feature extraction module of YOLOv5 is replaced by the richer C2f module based on gradient flow. This adjusts the number of channels for different scale models, thereby reducing model computation and improving convergence speed and convergence efficiency.

### 2.4 Verification of accuracy

The models were evaluated on the basis of three performance indicators: accuracy *P* (precision), *R* (recall rate), and *mAP* (mean average precision). The equations used to calculate *P*, *R*, and *mAP* are listed below:


(7) $$P=\frac{TP}{TP+FP}$$

(8) $$R=\frac{TP}{TP+FN}$$

The *F1*-score is expressed as in Equation 9:


(9) $$F1=\frac{2\times P\times R}{P+R}$$

(10) $$mAP=\frac{\sum_{i=1}^{n}AP_{i}}{n}$$

*TP* is the number of targets correctly detected by the model; *FP* is the number of targets incorrectly detected by the model; *FN* is the number of targets not detected by the model; and *n* is the number of categories.

The performance of each model in terms of counting sorghum seedlings was determined on the basis of the determination coefficient (*R^2^*), root mean square error (*RMSE*), and relative root mean square error (*RRMSE*) as evaluation metrics.


(11) $$R^{2}=1-\frac{\sum_{1}^{n}{\left(Y_{i}-X_{i}\right)}^{2}}{\sum_{1}^{n}{\left(Y_{i}-{\bar{Y}}_{i}\right)}^{2}}$$

(12) $$RMSE=\sqrt{\frac{\sum_{1}^{n}{\left(Y_{i}-X_{i}\right)}^{2}}{n}}$$

(13) $$\text{RRMSE}=\sqrt{\sum_{\text{i}=1}^{\text{n}}{\left({\text{Y}}_{\text{i}}-{\text{X}}_{\text{i}}\right)}^{2}\times \frac{1}{\text{n}}}\times \frac{1}{{\bar{\text{Y}}}_{\text{i}}}$$

Where Y_i_, ${\bar{Y}}_{i}$ , and X_i_ are the number of sorghum seedlings in the image marked by the *i*-th person, the average number of sorghum seedlings marked by the *i*-th person, and the predicted number of sorghum seedlings, respectively, and *n* is the number of test images.

## 3 Results

### 3.1 Identification and segmentation of sorghum seedlings from images

**Figure 4:**
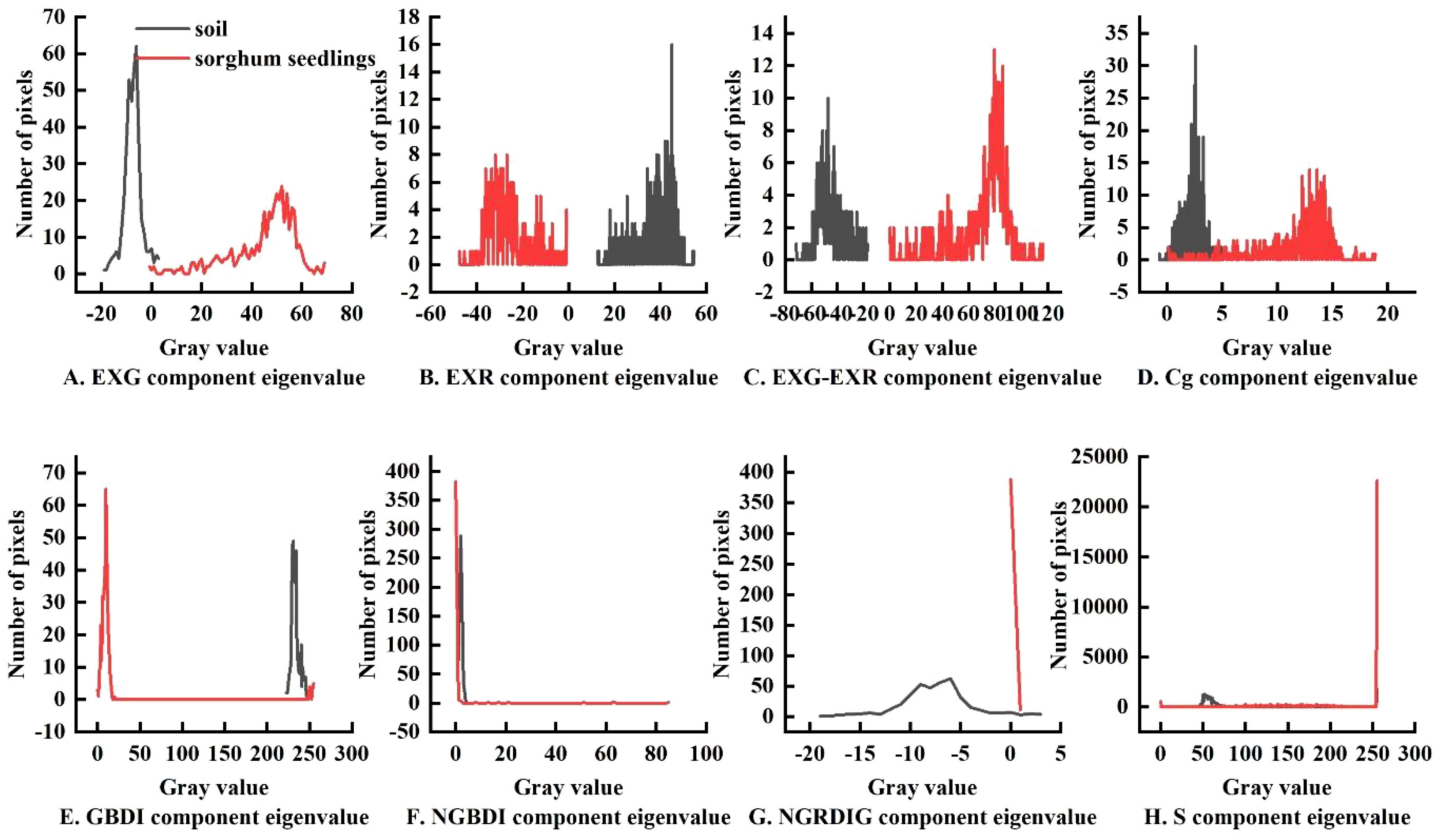
Color histogram of sorghum, soil in sorghum fields: **(A)** EXG index segmentation, **(B)** EXR index segmentation, **(C)** EXG-EXR index segmentation, **(D)** Cg index segmentation, **(E)** GBDI index segmentation, **(F)** NGBDI index segmentation, **(G)** NGRDI index segmentation, **(H)** S-component color segmentation.

To analyze the relationship between the sorghum target and background pixels under different color indices, 36 images of sorghum fields were selected for feature analysis. First, sorghum and soil sample points were extracted from the sorghum field images. The sample size was set to 20 × 20 pixels. Then, the sample images were converted using eight color indices, and the color histograms of sorghum plants and soil were statistically analyzed, as shown in **Figure 4**.

**Figure 5:**
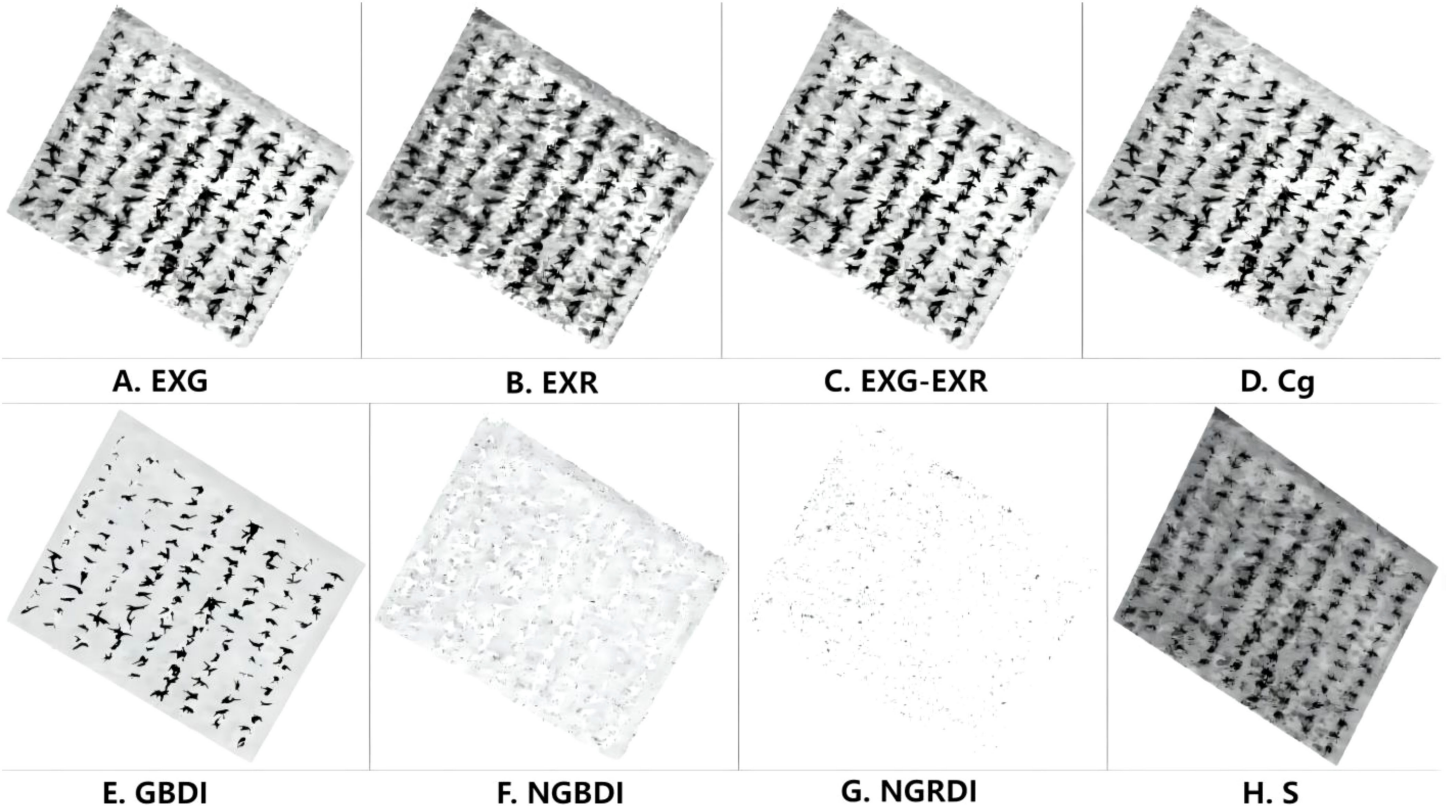
Otsu segmentation results under different color characteristics: **(A)** Threshold segmentation under EXG index, **(B)** Threshold segmentation under EXR index, **(C)** Threshold segmentation under EXG-EXR index, **(D)** Threshold segmentation under Cg index, **(E)** Threshold segmentation under GBDI index, **(F)** Threshold segmentation under NGBDI index, **(G)** Threshold segmentation under NGRDI index, **(H)** Threshold segmentation under EXG S-component.

As shown in **Figure 4A**, there was a clear separation between soil and sorghum using the EXG index, consistent with the Otsu threshold segmentation characteristic. As shown in **Figures 4D–H**, image segmentation using some of the other indexes resulted in common areas between the target, sorghum, and non-target (soil) areas. Therefore, if all the soil in the image was removed, some of the sorghum would also be removed, resulting in poor segmentation. As shown in **Figures 4B** and **C**, some color indices resulted in clear separation between soil and sorghum, but the areas were separated by large gaps with multiple segmentation points, resulting in poorer segmentation performance. A comparison of segmentation using all eight indexes is shown in **Figure 5**. Through comparative analysis, it was found that the segmentation effects of EXG, EXR, EXG-EXR, and Cg were good. Analyses of the actual segmentation results revealed that the sorghum target segmentation was less contaminated and more complete when using the EXG color index. Therefore, we selected the EXG color index for sorghum target extraction and segmentation.

### 3.2 Comparison of machine learning and deep learning model analysis

**Figure 6:**
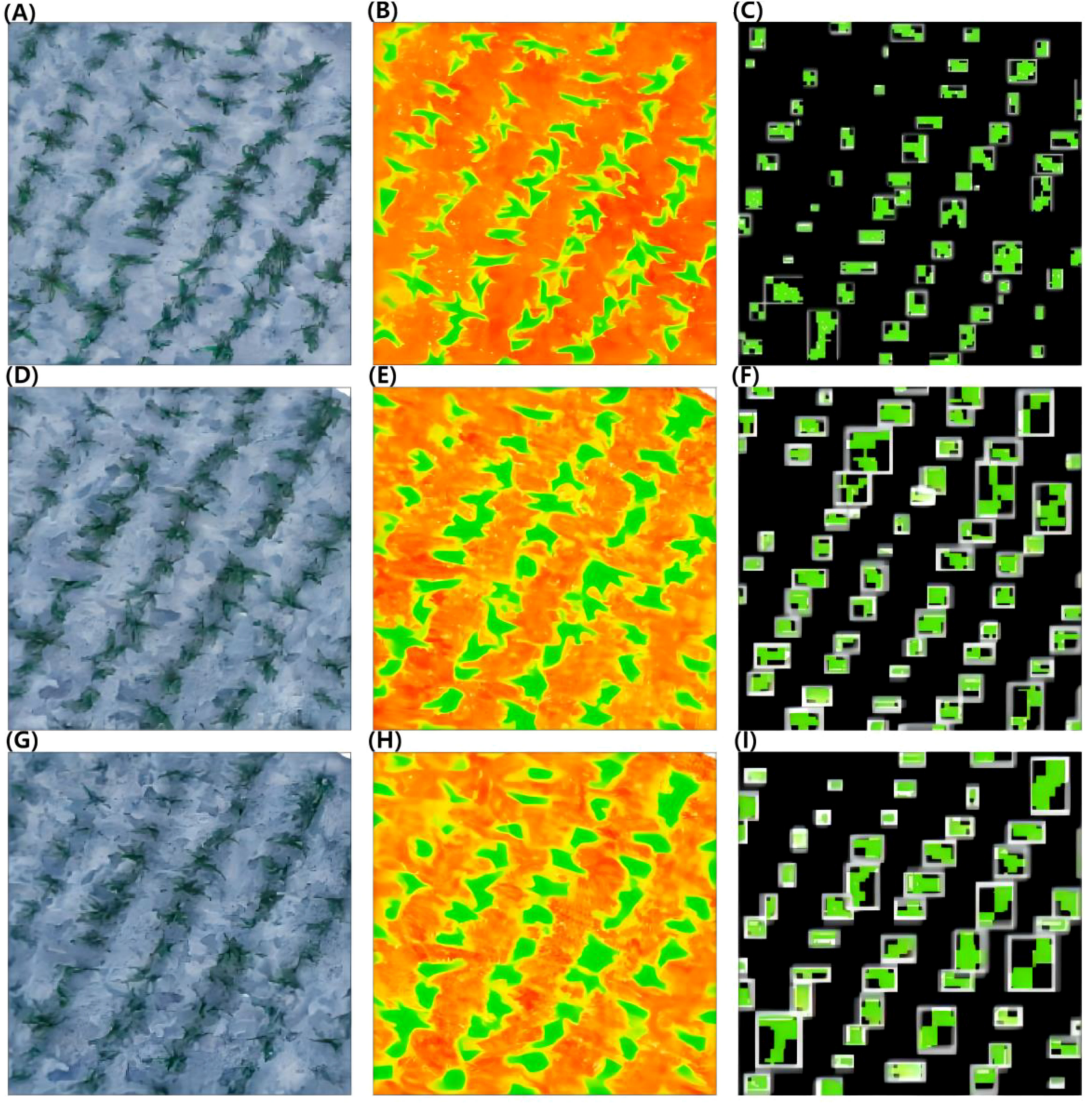
SVM model detection and counting of sorghum seedlings. **(A, D, G)** Original images at heights of 15 m, 30 m, and 45 m. **(B, E, H)** The segmentation results of EXG at heights of 15 m, 30 m, and 45 m. **(C, F, I)** The recognition results of SVM at heights of 15 m, 30 m, and 45 m.

An example of SVM detection and counting of the sorghum seedlings from the dataset is shown in **Figure 6**. **Figures 6A, D, G** show the original images captured at heights of 15 m, 30 m, and 45 m, **Figures 6B, E, H** show the segmentation results using the EXG index at heights of 15 m, 30 m, and 45 m, and **Figures 6C, F, I** show the recognition results obtained using the SVM algorithm for images captured at heights of 15 m, 30 m, and 45 m. In the images captured at 15 m, 30 m, and 45 m, manual counting identified 121, 126, and 125 sorghum seedlings, respectively, while the SVM algorithm detected 120, 127, and 112 seedlings, respectively.

**Figure 7:**
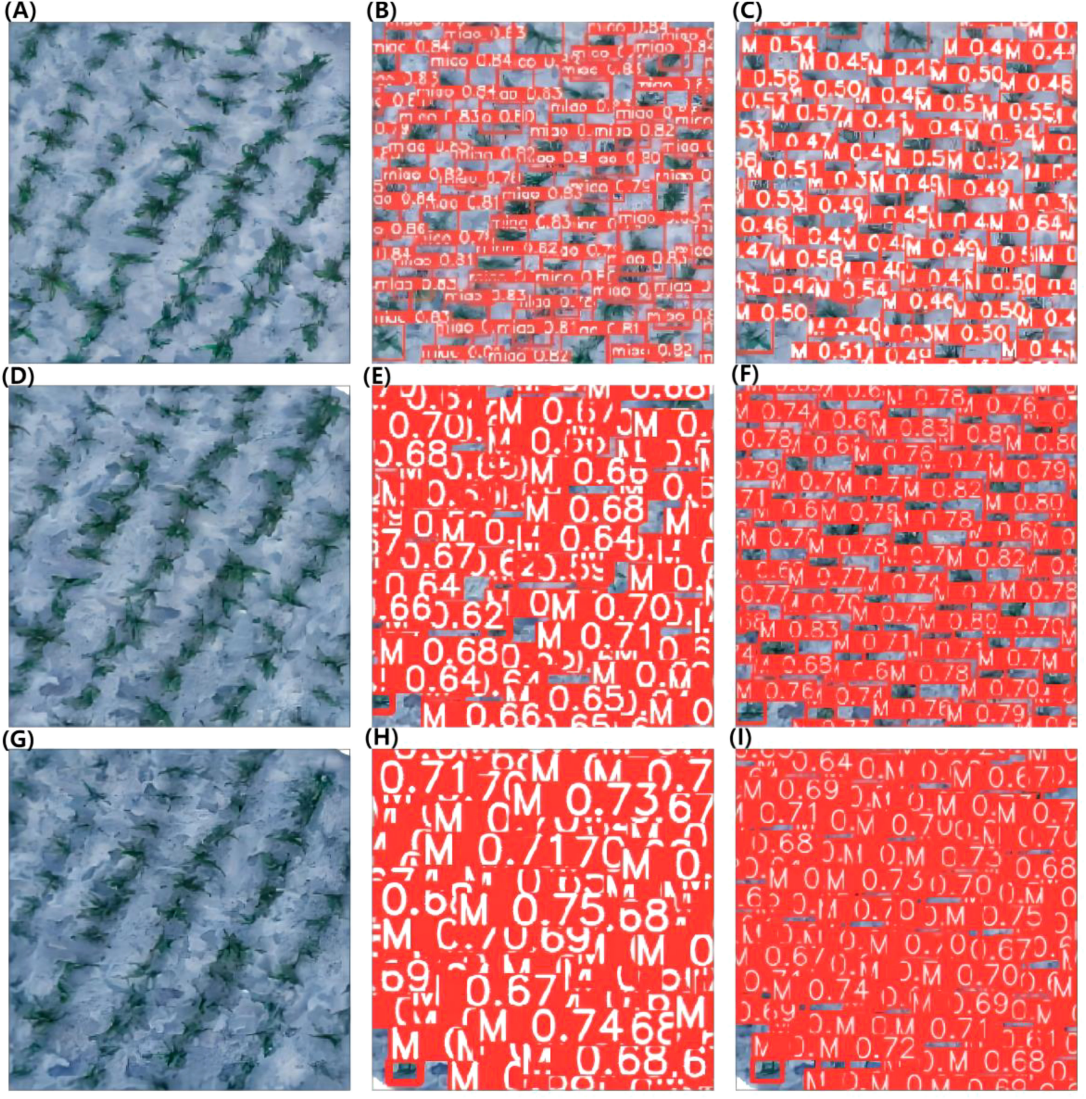
YOLOv5 and YOLOv8 model detection and counting of sorghum seedlings. **(A, D, G)** Original images at heights of 15 m, 30 m, and 45 m. **(B, E, H)**: The recognition effect diagrams of YOLOv5 at heights of 15 m, 30 m, and 45 m. **(C, F, I)** The recognition effect diagrams of YOLOv8 at heights of 15 m, 30 m, and 45 m.

**Figure 7** shows examples of the use of YOLOv5 and YOLOv8 to detect and count sorghum seedlings from the dataset. **Figures 7A, D**, **G** show the original images captured at heights of 15 m, 30 m, and 45 m, respectively. **Figures 7B, E, H** show the recognition effect diagrams of YOLOv5 at heights of 15 m, 30 m, and 45 m, respectively. **Figures 7C, F, I** show the recognition effect diagrams of YOLOv8 at heights of 15 m, 30 m, and 45 m, respectively. Visual counting from images captured at heights of 15 m, 30 m, and 45 m indicated that there were 121, 126, and 125 sorghum seedlings, respectively. YOLOv5 and YOLOv8 detected 118 and 123 sorghum seedlings, respectively, in images captured at 15 m height; 110 and 122 sorghum seedlings, respectively, respectively, in images captured at 30 m height; and 112 and 125 sorghum seedlings, respectively, in images captured at 45 m height. The background of the tested sorghum field images was very complex, with partial overlap, object occlusion, and soil agglomerates. All of these factors can reduce the accuracy of predictions, so the algorithm must be very effective to counter these sources of error. Despite the difficult background, the experimental results were still very accurate and achieved the experimental purpose.

The performance evaluation results of sorghum seedling detection based on SVM, YOLOv5, and YOLOv8 algorithms are shown in **Table 3**. For the images collected at three heights, the results predicted using YOLOv8 had the highest *R^2^* and the lowest *RMSE*; while the results predicted using SVM had the lowest *R^2^* and those predicted using YOLOv5 had the highest *RMSE*. According to the evaluation criteria, the larger the *R^2^* value and the smaller the *RMSE*, the better the model. Our results show that deep learning was superior to machine learning for analyses of these image data. The YOLOv8 model showed good recognition of sorghum seedlings in images collected at different flight altitudes.

**Table 3:** Model performance evaluation.

Models		Performance evaluation								
		P	R	mAP@0.5	F1	R^2^	RMSE	RRMSE	Time of training (min)	Time of testing (sec)
15m	SVM	–	–	–	–	0.67	1.16	0.95%	2.19	6
	YOLOv5	0.88	0.88	0.86	0.88	0.76	1.33	1.09%	230. 40	61
	YOLOv8	0.85	0.87	0.80	0.86	0.93	0.12	0.10%	241. 80	72
30m	SVM	–	–	–	–	0.57	6.32	5.26%	2.16	5
	YOLOv5	0.78	0.82	0.79	0.80	0.57	14.77	12.31%	190.70	42
	YOLOv8	0.90	0.91	0.89	0.90	0.90	0.40	0.33%	198.90	51
45m	SVM	–	–	–	–	0.51	5.99	4.95%	2.05	4
	YOLOv5	0.86	0.89	0.89	0.79	0.56	7.71	6.37%	178.60	36
	YOLOv8	0.95	0.97	0.96	0.96	0.71	2.82	2.33%	185.40	43

**Figure 8:**
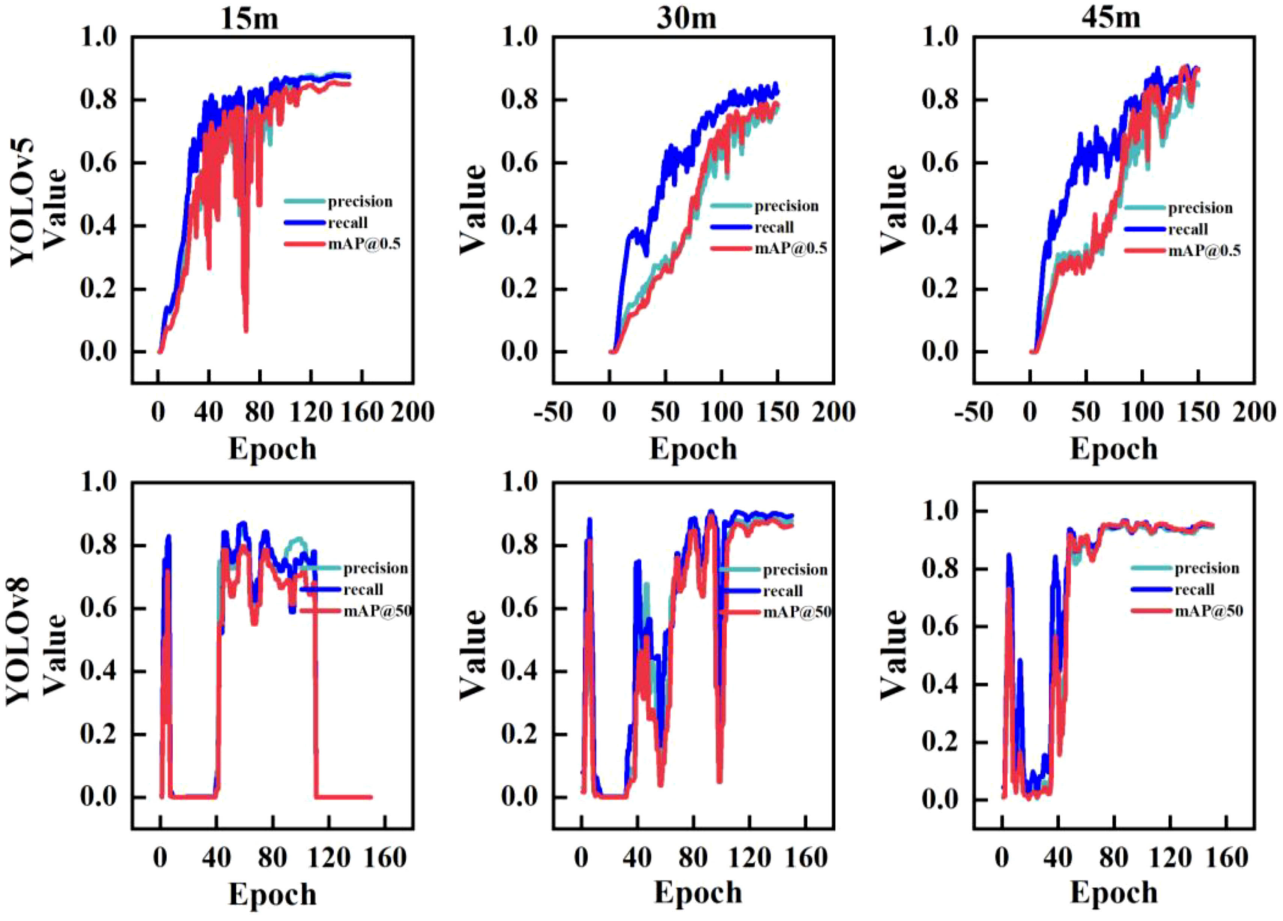
YOLOv5 and YOLOv8 Model results. The Epoch refers to one complete traverse of the model over the entire training dataset.

When training YOLOv5 and YOLOv8 networks after dividing the dataset, the Epoch was set to 150. The Epoch refers to one complete traverse of the model over the entire training dataset, and increasing its value can improve the performance of the model. However, it may result in overfitting if it is increased too much. **Figure 8** shows the changes in the *P*, *R*, and *mAP* of the YOLOv5 and YOLOv8 network models as the Epoch value was increased during training. As shown in the figure, the *P*, *R*, and *mAP* of the YOLOv5 model outputs based on images collected at three different heights tended to stabilize after Epoch=100, whereas those of the YOLOv8 model outputs tended to stabilize after Epoch=120.

**Figure 9:**
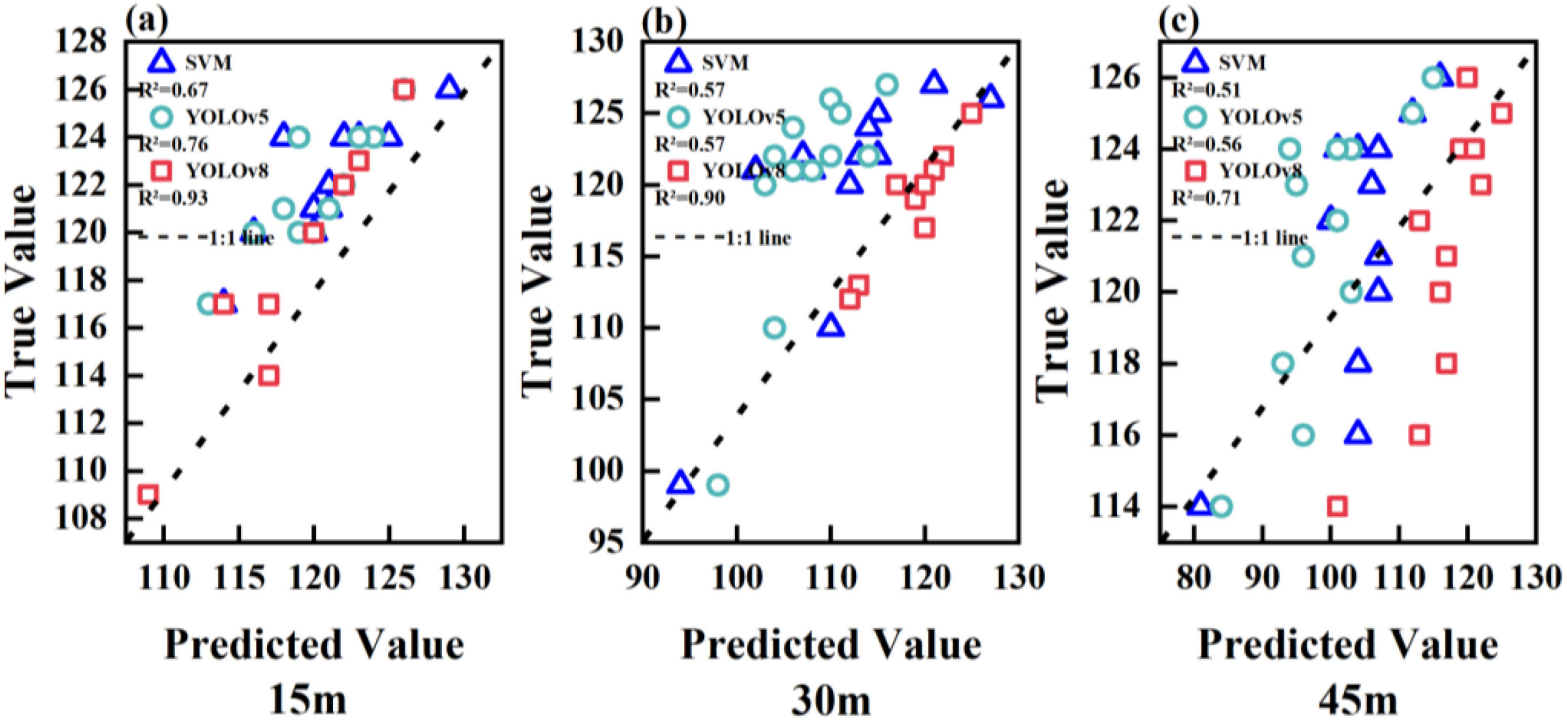
The R^2^ results of SVM, YOLOv5, and YOLOv8 at heights of 15 m, 30 m, and 45 m: **(A)** The R^2^ results for the three models at a height of 15 m, **(B)** The R^2^ results for the three models at a height of 30 m, **(C)** The R^2^ results for the three models at a height of 45 m.

**Figure 9** shows a fitting analysis of the predicted values of the number of sorghum seedlings in the validation set using the SVM, YOLOv5, and YOLOv8 models against the actual number of sorghum seedlings. For the results predicted using the SVM, YOLOv5, and YOLOv8 models, at a height of 15 m, the *R^2^* values were 0.67, 0.76, and 0.93, respectively; at a height of 30 m, the *R^2^* values were 0.57, 0.57, and 0.90, respectively; and at a height of 45 m, the *R^2^* values were 0.51, 0.56, and 0.71, respectively. These results show that, as the height of image capture increased, the *R^2^* of the three models decreased.

### 3.3 Estimation of the number of seedlings

**Figure 10:**
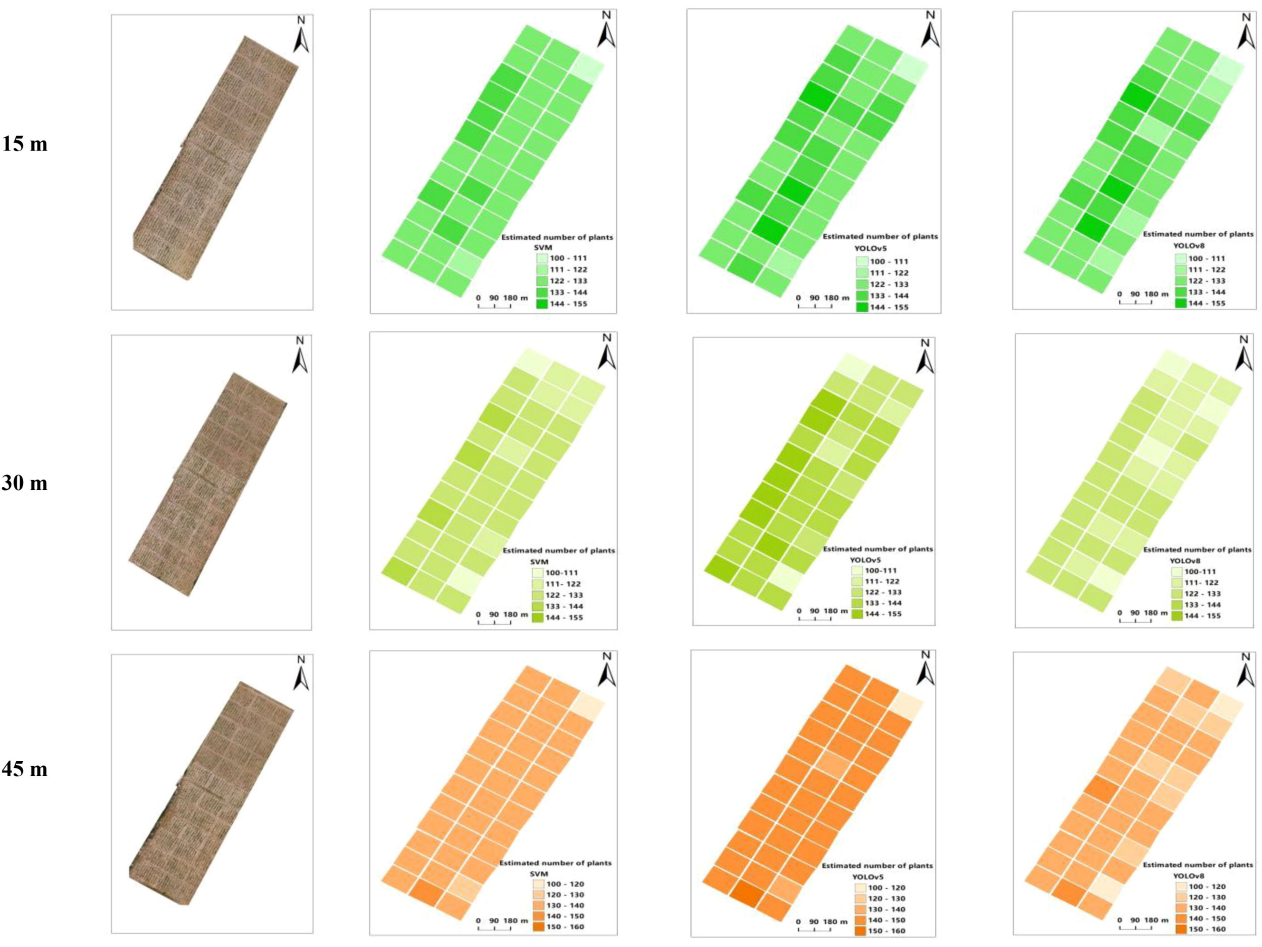
Estimation of the number of seedlings emerging.

The number of sorghum seedlings estimated using the SVM, YOLOv5, and YOLOv8 models was plotted as the number of seedlings per plot (**Figure 10**). By comparing and analyzing the SVM, YOLOv5, and YOLOv8 models, we found that the YOLOv8 algorithm provided the most accurate estimate of seedling number from images captured at three different flight altitudes, compared with the actual (manually counted) number. The SVM and YOLOv5 models overestimated the number of sorghum seedlings, possibly because of duplicate detection of some leaf information.

## 4 Discussion

Presently, numerous scholars have utilized low-altitude aerial remote sensing technology employing drones for the purpose of estimating crop quantities (Zhao et al., 2018; Zhou et al., 2018; Fu et al., 2020). Fu et al. (2020) employed drone-based remote sensing imagery along with an object-oriented multi-scale segmentation algorithm for automated extraction of sword grass plant counts, yielding a recognition accuracy of 87.1%. While this approach demonstrates favorable segmentation outcomes and straightforward implementation, it is important to note that the use of multi-scale segmentation may lead to over-segmentation, potentially impacting the precision of plant count recognition. Zhou et al. (2018) converted the RGB images captured by drones to grayscale images, applied threshold segmentation to separate corn seedlings from the soil background, and subsequently extracted the skeleton of the corn seedlings for counting purposes. The correlation between the recognition results and manual counting ranged from 0.77 to 0.86. However, this method of crop quantification through skeleton extraction or multivariate linear fitting exhibits limited estimation accuracy. Zhao et al. (2018) employed thresholding for rapeseed plant segmentation and extraction of area, aspect ratio, and ellipse fitting shape features, subsequently establishing a regression model correlating with the number of seedlings. The R-squared values for the regression models were 0.845 and 0.867. However, this image processing method utilizing shape features combined with thresholding is susceptible to influences such as plant occlusion, weed interference, and variations in light intensity, thereby limiting its general applicability. This study employed color features for the extraction and estimation of sorghum targets. The findings indicated that: (1) The combination of the EXG color index with the Otsu threshold segmentation method accurately and completely segmented the background and plant targets in the image. (2) Estimation of the plant target using SVM resulted in a high false positive rate and a high root mean square error. Therefore, threshold segmentation and machine learning algorithms for crop count estimation are most suitable for scenarios with limited sample sizes or when rapid identification results are imperative.

The YOLO series of single-stage object detection, characterized by faster processing speeds, has demonstrated its efficacy in the detection and quantification of crop targets in complex environments. Zhao et al. (2021a) enhanced the accuracy of wheat spike detection in drone images by incorporating additional detection layers into YOLOv5 and optimizing the confidence loss function, resulting in an average detection accuracy of 94.1% for identifying small and highly overlapping wheat spikes. Barreto et al. (2021) utilized unmanned aerial vehicles equipped with RGB cameras and employed deep learning image analysis techniques to achieve fully automated counting of sugar beet, corn, and strawberry seedlings. The identified crop numbers were compared with ground truth data, resulting in an error rate of less than 5%. Korznikov et al. (2023) utilized YOLOv8 for the identification of two perennial coniferous species, achieving an overall accuracy of 0.98. This study utilized deep learning models algorithm (YOLOv5, YOLOv8) for the extraction and estimation of sorghum targets. The findings revealed that: (1) At a height of 15m, YOLOv5 achieved a detection accuracy of 0.76, while YOLOv8 reached 0.93. (2) At heights of 30m and 45m, the accuracy and recall rates of YOLOv8 on the test set were higher by 15.380% and 10.980% compared to those of YOLOv5 at 30m, and by 10.470% and 8.990% at 45m, respectively. (3) The prediction errors for the number of emerged seedlings at three different flight heights were observed as follows: for YOLOv5: 1.630%, 12.070%, and 7.650%; for YOLOv8: 0.020%, 0.10%, and 2.95%. This indicates that seedling detection is a challenging task if images are collected at different heights.

Therefore, the effect of the flight altitude of UAV on the detection of plant seedling numbers using the various models was significant. The altitude at which a UAV flies has a direct impact on the quality and accuracy of the data it acquires. Images captured at lower flight altitudes have higher spatial resolution, so lower heights allow UAV to capture more detailed information, which is critical for identifying and counting plant seedlings.

The model used to process the data acquired by the UAV will affect the final detection results. Some studies have used vegetation index models such as the Normalized Difference Vegetation Index to estimate biomass or leaf area index, but these models are subject to saturation when estimating plant populations, and that limits their accuracy (Zhang et al., 2023a). Therefore, it is important to develop and test new models, to improve the application of UAV in plant population detection. Both the flight altitude and model selection need to be fine-tuned to the specific characteristics of the farmland and the monitoring objectives. Errors in the labeling process can also affect the accuracy of the model. For instance, some sorghum seedlings might not be labeled correctly, leading to false negatives or positives. On the other hand, image processing time is a critical aspect of machine learning and deep learning algorithms. The training time for deep learning models is significantly longer than that for SVM models. The training time for deep learning models primarily depends on factors such as the number of images used and the hardware utilized.

In summary, in this study, the performance of deep learning algorithms significantly surpassed that of a traditional machine learning algorithm, consistent with the results of another study (Tan et al., 2022). Deep learning algorithms can be used for real-time monitoring of field-grown seedlings, particularly in large-scale planting scenarios (García-Santillán et al., 2017). Our results provide a technical reference for using images from UAV, combined with an appropriate model, to count field-grown sorghum seedlings. This has potential applications in precision breeding, phenotype monitoring, and yield prediction of sorghum. However, the complexity of the YOLO network is still high, and the image quality needs to be improved. In future research work, it will be important to develop a high-performance, lightweight network and optimize image quality to achieve accurate real-time detection.

## 5 Conclusions

We tested three detection models to count the number of sorghum seedlings in UAV images captured at different heights. The three models were a machine learning approach (SVM) and two deep learning approaches (YOLOv5 and YOLOv8). The main results were as follows:

1. The *R^2^* of the three methods decreased as the height of image capture increased. The *R^2^* values for results obtained from images captured at heights of 15 m, 30 m, and 45 m were, respectively, (SVM: 0.67, 0.57, 0.51), (YOLOv5: 0.76, 0.57, 0.56), and (YOLOv8: 0.93, 0.90, 0.71).
2. On the basis of the accuracy, recall, and *F1* scores of the YOLO series, the detection performance of YOLOv8 was better than that of YOLOv5 with the increase in drone altitude.
3. The YOLO algorithm outperformed the SVM algorithm. The YOLOv8 model was more suitable for identifying and estimating the number of sorghum seedlings in a field.

The results demonstrated that the proposed method can be effective for large-scale counting of sorghum seedlings. Compared with field observation by humans, the UAV-based approach can obtain growth information of crop seedlings more efficiently and accurately, so it is an important method for precision agriculture under field conditions.

## Data Availability

The raw data supporting the conclusions of this article will be made available by the authors, without undue reservation.
